# 3D histological mapping of hippocampal subfields: a comparative study in patients with schizophrenia and healthy controls

**DOI:** 10.3389/fpsyt.2025.1682782

**Published:** 2025-10-06

**Authors:** Halil İbrahim Öztürk, İmren Kurt, Gizem İskender, Süleyman Dönmezler

**Affiliations:** ^1^ Department of Psychiatry, SANKO University, School of Medicine, Gaziantep, Türkiye; ^2^ Department of Psychiatry, Başakşehir Çam and Sakura City Hospital, Istanbul, Türkiye; ^3^ Department of Psychiatry, Istanbul Prof. Dr. Cemil Tascioglu City Hospital, Istanbul, Türkiye

**Keywords:** schizophrenia, hippocampus, 3D histological mapping, GPU computing, biomarkers, neuroimaging

## Abstract

Hippocampal deviations are a hallmark of schizophrenia, yet their regional specificity remain unclear. Neuroimaging studies have reported smaller volumes for each hippocampal subfields in schizophrenia compared to healthy controls but affected regions differ between studies. These conflicting findings highlight substantial heterogeneity within psychosis, which may be elucidated through more detailed sub-regional analyses. In this study, we aimed to determine whether patients with schizophrenia exhibit distinct volumetric alterations in specific hippocampal subfields compared to healthy controls. We analysed T1-weighted MRI data from the MCICShare project, employing the ComBat algorithm to harmonize data across multiple MRI platforms. Hippocampal subfields were segmented and quantified using the “Bayesian Segmentation with Histological Atlas”. All computational analyses were performed on Google Colab Pro+ with Nvidia A100 GPUs. Multiple ANCOVAs were then conducted, with diagnosis as the independent variable and each hippocampal subfield volume as the dependent variable, controlling for sex, age, and estimated intracranial volume. To mitigate type I error inflation, a 5% false discovery rate (FDR) threshold was applied. After excluding segmentation errors, we included 108 patients with schizophrenia and 94 healthy controls in the final analysis. Among the examined subfields, only the right CA2 showed a significant volumetric difference after FDR adjustment (F = 8.562, P_FDR_ =0.048, η^2^p=0.042). Our findings underscore the value of high-granularity segmentation approaches and highlight the potential importance of CA2 alterations in schizophrenia’s pathophysiology, thereby guiding future research directions and clinical applications.

## 1 Introduction

Emil Kraepelin, initially characterized schizophrenia as a progressive condition leading to cognitive decline, and termed this disorder dementia praecox (1). This nomenclature was later revised by Eugen Bleuler, who introduced the term “schizophrenia” that is widely recognized today (2). Both Kraepelin and Bleuler emphasized the organic and “tangible” aetiologies of the disorder, attributing the pathology to toxic and anatomical damage to cortical cells, rather than to “psychic” influences (3). Their perspectives have significantly influenced the understanding and treatment of the disorder since that time.

This complex neuropsychiatric disorder, with a global lifetime prevalence of roughly 0.75% (4) and contributing to reduced lifespan, is characterized by a multifaceted aetiology. Insights into its neurodevelopmental origins have emerged from early neurobehavioral indicators observed in the children of affected parents, suggesting that the disorder may originate during early developmental stages (5–7). The structural brain abnormalities observed in patients with schizophrenia, including thinning of the cerebellar cortex (8) and alterations in the thalamus (9), and striatum (10) highlight the disorder’s profound impact on brain structure. Notably, the hippocampus, essential for memory and other cognitive functions, shows significant morphological and functional deviations, underscoring its critical role in the pathology of schizophrenia (11). Recent insights suggest the importance of examining specific hippocampal subfields, particularly the anterior region, for a more nuanced understanding of volume changes related to psychosis (12).

The hippocampal formation comprises three primary sections: the hippocampus proper (alternatively called Ammon’s horn or cornu ammonis), the dentate gyrus, and the subiculum (referred to as the subicular cortex) (13). The Cornu Ammonis (CA) defines the different layers of the hippocampus and there are four hippocampus subfields (CA1, CA2, CA3 and CA4) (14). The hippocampus, a central element of the brain’s limbic system, plays a critical role in learning, memory, and emotional regulation. It comprises several distinct subfields, each contributing uniquely to its overall function: I) CA1 Region: Essential for the consolidation of long-term memories (15), II) CA2 Region: Plays a role in social memory and behaviors, distinguishing it from other hippocampal areas (16), III) CA3 region of the hippocampus plays a crucial role in the swift encoding of memories (17), IV) and the CA4 region is an important anatomic crossroad for innervation by perforant and mossy fiber pathways connecting hippocampus with several other sites in the brain (18). The dentate gyrus, as the primary recipient of sensory input from the entorhinal cortex, serves as the initial processing stage for episodic memory formation, uniquely processing and transmitting information to the hippocampal CA3 field due to its distinct neuroanatomy (19). Besides being a recipient of hippocampal inputs, the subiculum is crucial for learning and memory, particularly through its unique projections to the anterior thalamic nuclei that facilitate the resolution of complex memory tasks (20).

The role of the hippocampus in schizophrenia is not yet fully understood. Neuroimaging studies have reported smaller volumes for each hippocampal subfields in schizophrenia compared to healthy controls but affected regions differ between studies. (21). The studies evaluating the relationship between hippocampal subfield volumes and symptomatology have also reported varied results. Kühn et al. (22) reported that patients with stronger positive symptoms had smaller CA2/3 and CA1 subfields. It has also been reported that positive psychotic symptoms, defined by hallucinations and delusions, are associated with CA1 deformity, CA1 contraction, larger CA1 volume, and smaller CA2/3, CA4/DG, presubiculum, and subiculum volumes. For negative psychosis symptoms, smaller CA2/3 and CA4/DG volumes, smaller subicular volume and subicular contraction have been associated in different studies (21). Cognitive problems, one of the core areas of impairment in schizophrenia, have been examined in a small number of studies related to hippocampal subfields morphometry, and these studies highlight the lower subiculum volumes (21, 23).

The structure, tight connections, and functions of the hippocampus are quite complex therefore there is no clear consensus on the delimitation of hippocampal subfields according to segmentation protocols. Research on the refinement and validation of the hippocampal subfields’ methods are also actively ongoing; therefore, it is recommended that preliminary findings be interpreted with caution (24). In addition to volumetric reductions, morphological abnormalities in the hippocampus are also known in schizophrenia, including incomplete inversion patterns (IHI) and inward deformations (11). IHI can challenge the performance of automated hippocampal segmentation methods (25, 26).

Recent research employing the automated hippocampal segmentation technique developed by Iglesias et al. (27) has demonstrated that individuals with schizophrenia exhibit significantly reduced volumes in the bilateral CA1 and molecular layers compared to healthy controls (28). The team, also responsible for this initial segmentation technique, has introduced the NextBrain, a next-generation tool for 3D histological mapping (29). This advanced method enables highly detailed examination of hippocampal subfields *in vivo*, which we employ in our current study for further analysis.

Our study leverages this advanced method to explore the intricate anatomy of the hippocampus in living individuals, aiming to identify significant structural deviations in individuals with schizophrenia compared to healthy controls. We hypothesized that such detailed examination will reveal volume reductions in hippocampal subfields in patients with schizophrenia compared to healthy controls, which may serve as biomarkers for the disorder and therapeutic targets.

## 2 Materials and methods

The study was conducted in accordance with the ethical principles of the Declaration of Helsinki and approval for the study was granted by the SANKO University Non-Interventional Research Ethics Committee on 27/08/2024, with no: 2024/8.

### 2.1 Study design

In our cross-sectional study, we investigated patients diagnosed with schizophrenia using magnetic resonance imaging (MRI) data obtained from the MCICShare study repository. Following the reconstruction process, we conducted a comparative analysis to examine the differences in hippocampal sub-regions volume measurements in these patients and control group.

### 2.2 Data collection

SchizConnect (http://schizconnect.org/) is an open-access repository for neuroimaging data, consisting of MRI scans from schizophrenia subjects and healthy controls collected across multiple research studies. Our study utilizes the MCIC Collection project (30) to investigate volumetric differences hippocampal subfields among individuals with schizophrenia and healthy controls. We obtained the MRI images from schizconnect.org through online data requests. Participants in these projects undergo extensive baseline evaluations, including T1-weighted MRI acquisitions and systematic clinical assessments.

For the T1-weighted MRI scans utilised in this study, the imaging parameters varied based on the scanner’s magnetic field strength. For 3T scanners, the repetition time (TR) was set at 2530 ms, echo time (TE) at 3.79 ms, flip angle (FA) at 7, inversion time (TI) at 1100, and bandwidth at 181. For 1.5T scanners, the TR was 12 ms, TE 4.76 ms, FA 20, and bandwidth 110. Both scanner types shared a voxel size of 0.625 × 0.625 mm and a slice thickness of 1.5 mm. The field of view (FOV) was established at a 256 × 256 ×128 cm matrix, with a baseline FOV of 16 cm, which could be increased to 18 cm for full brain coverage. In terms of site-specific equipment, site A used a 1.5 T Siemens Sonata for all structural imaging, while site C employed a 3T Siemens Trio. Site D conducted structural imaging using a 1.5T Siemens, and site B performed all structural scans with a 1.5T GE SIGNA. This overview provides a concise summary of the structural imaging parameters used in our study. For detailed information on the imaging protocols readers are encouraged to refer to the original data publication (30).

### 2.3 Study participants

Our query included all anatomical images of participants classified as “Schizophrenia Broad” within MCICShare project. This search yielded imaging results and clinical characteristics of 204 subjects (HCs: healthy controls, SCZ: patients with schizophrenia). The distribution of these subjects across different sites was as follows: Site A (n =88 [HCs =44/SCZ =44]), Site C (n =58 [HCs =26/SCZ =32]), Site D (n=58 [HCs =25/SCZ =33]).

### 2.4 Volumetric segmentation of hippocampal formation into its sub-regions

We employed the ‘full’ Bayesian version of the “Bayesian Segmentation with Histological Atlas ‘NextBrain’” (31). This software suite also incorporates algorithms from previous studies (32–34), and is integrated within the Freesurfer image analysis framework (http://surfer.nmr.mgh.harvard.edu/). Computational analyses were conducted using a paid subscription to Google Colab Pro+ with Nvidia A100 GPUs and a paid Google Drive plan.

In order to ensure quality control of segmentation, we utilized Freeview, a FreeSurfer-embedded program designed for viewing and manipulating structural anatomical scans in multiple planes and 3D, allowing us to create and edit layered volumes on the original scans, manually inspect segmented hippocampi from various orientations, visually assess the segmentation quality, identify inconsistencies, and make any necessary adjustments.

### 2.5 Variables

**Figure 1:**
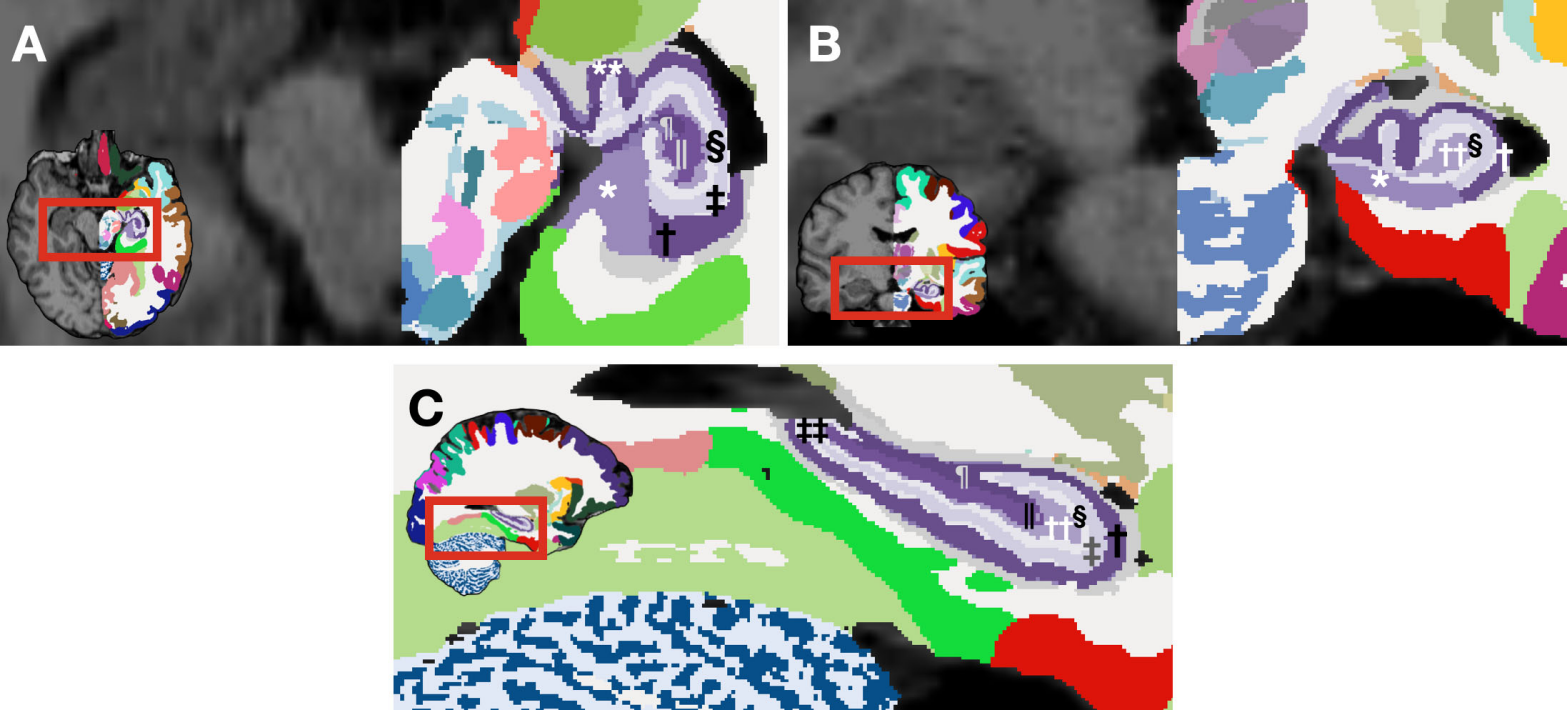
Segmented hippocampal subfields generated by NextBrain (29)for a representative subject from our dataset. Axial **(A)** and coronal **(B)** view of a segmented brain image: the left hemisphere is labeled according to NextBrain's lookup table, while the right hemisphere remains unlabeled for comparison. **(C)** Sagittal view of a segmented brain image. *rostral subiculum, †stratum pyramidale of rostral CA1, ‡stratum lacunosum moleculare of rostral CA1, § molecular layer of rostral DG, || pyramidal cells of rostral CA4, ¶ stratum pyramidale of rostral CA3, ** stratum pyramidale of uncal CA1, †† polyform layer of rostral dentate gyrus, ‡‡ stratum lacunosum moleculare of caudal CA1.

The primary explanatory variable was diagnosis: binary categoric as the independent variable, contrasting healthy controls with schizophrenia patients. Primary dependent variables were hippocampal subfields volumes. While the NextBrain can segment hippocampus into numerous subfields (several of which are illustrated in **Figure 1**), our analyses focused on the primary subfields of interest; CA1, CA2, CA3, CA4, the subiculum, and the dentate gyrus; by aggregating and summing the fine-grained segmentation outputs. We also collected demographic data (age, gender, education, e.g.) and clinical characteristics (drug-naive status [TRUE/FALSE], duration of illness, etc.) to account for potential confounding factors in our study.

### 2.6 Statistical methods

Continuous variables were presented as means (standard deviation) if normally distributed, and as medians [25^th^–75^th^ percentile] otherwise. Between-group comparisons for continuous variables were performed using t-tests for normal distributions and Mann-Whitney U test for non-normal distributions. Categorical variables were expressed as frequencies and percentages, with Pearson’s χ² test or Fisher’s exact test employed according to theoretical frequency thresholds.

Technical variability is a significant problem in multicenter neuroimaging studies. There are two main types of technical variability: density unit effects and scanner effects. Scanner-related effects can be eliminated with image-level and regional-level harmonization techniques (35). In this study, we applied the ComBat batch-adjustment algorithm (36–38) to harmonize hippocampi volumes and estimated intracranial volume (eTIV) across three distinct MRI platforms. After post-harmonization analyses, variability attributable to MRI scanners for hippocampal sub-region volumes was not significant, indicating successful mitigation of site-related bias.

We favoured univariate models (multiple ANCOVAs) over multivariate approaches (MANCOVA) (39) to emphasize metrics at the sub-regional level, thereby facilitating more precise localization of structural differences. Each ANCOVA modelled hippocampal sub-regions as dependent variables, controlling for sex, age, and eTIV. Prior to performing ANCOVAs, we verified that standard assumptions, including normally distributed residuals, homogeneity of variances, linear relationships between covariates and dependent variables, and homogeneity of regression slopes, were met. To mitigate type I error inflation associated with multiple comparisons, the false discovery rate (FDR) was controlled using the Benjamini-Hochberg procedure, with the FDR set at 5%.

All statistical tests were two-tailed, with an α error of up to 5% considered acceptable to define the statistical significance of any results. All analyses were conducted using R software version 3.6.0 (R Core Team, 2019; R Foundation for Statistical Computing, Vienna, Austria). To implement the ComBat batch-adjustment algorithm, we utilized the *sva package* (40), univariate analyses and ANCOVAs were performed with base R functions and the *car package* (41), adjustments for multiple comparisons via the Benjamini-Hochberg false discovery rate were carried out using the *stats package’s* built-in p.adjust function, and all visualisations were created using Keynote version 13.0 (7036.0.126) (Apple Inc., Strobe Inc. – [SproutCore]).

## 3 Results

### 3.1 Demographics and clinical characteristics

Initially, our study included 116 schizophrenia patients and 96 healthy controls. Due to segmentation errors in the NextBrain pipeline affecting 8 patients and 2 control in the MCICShare dataset, our final analysis was conducted with 108 patients and 94 controls. The patient group had a mean age of 34.5 (± 11.1) years and was predominantly male. The healthy control group had a mean age of 33.2 (± 12.2) years, with a male predominance as well. A comprehensive descriptive analysis can be found in **Table 1**.

**Table 1:** Demographics and clinical characteristics of the participants of the MCICShare.

Variable	HCs n = 94	Schizophrenia n = 108	Test	*P*
Age, years	33.2 (12.2)	34.5 (11.1)	0.788*	0.432
Sex, female	30 (31.9%)	26 (24.1%)	1.54^†^	0.214
Highest education achieved, years	15.5 (2.47)	12.9 (2.64)	7.06*	**<.001**
Handedness, right	86 (91.5%)	94 (87.0%)	3.69^†^	0.297
Years of schizophrenia	—	7 [2 - 19]	—	—
Positive symptoms	—	5 [3 - 7]	—	—
Negative symptoms	—	7 [5 - 10]	—	—
Disorganised symptoms	—	1 [0 - 2]	—	—
e-TIV	1.61e+6	1.57e+6	2.10*	**0.037**
Neuroleptic naïve	—	5 (4.9%)	—	—

Data are presented as mean (± standard deviation) or median [25th percentile - 75th percentile] for continuous variables and number (percentage) for categorical variables.

*independent samples t-test, † contingency tables X2 test.

HCs, healthy controls; eTIV, estimated total intracranial volume.statistically significant values ​​are shown in bold.

### 3.2 Multiple ANCOVAs

Following multiple ANCOVAs, which controlled for sex, age, and eTIV, and subsequent FDR adjustments, the right hemisphere CA2 region exhibited a significant effect (F = 8.562, P_FDR_ = 0.048, η^2^p = 0.042) (**Table 2**).

**Table 2:** Results of multiple ANCOVAs assessing the effect of diagnosis on hippocampal subfield volumes, controlling for sex, age, and eTIV.

Dependent variable	Mean difference	F	P*
Subiculum - lh	8.18	0.751	0.668
CA1 - lh	13.8	0.741	0.668
CA2 - lh	6.87	4.353	0.120
CA3 - lh	8.81	4.569	0.120
CA4 - lh	1.03	0.440	0.746
Dentate gyrus - lh	6.21	1.071	0.668
Subiculum - rh	1.99	0.055	0.815
CA1 - rh	4.14	0.066	0.815
CA2- rh	9.69	8.563	**0.048**
CA3 - rh	7.54	4.289	0.120
CA4 - rh	0.73	0.341	0.746
Dentate gyrus - rh	1.81	0.117	0.815

Values considered statistically significant are denoted in bold.

*All reported P values have been adjusted using the Benjamini-Hochberg procedure to control the false discovery rate.

CA, Cornu ammonis, lh, left hemisphere, rh, right hemisphere.

## 4 Discussion

The main findings of this study suggest that, among the left hemisphere hippocampal subfields examined, no significant volumetric differences emerged after adjusting for multiple comparisons. Similarly, for most right hemisphere subfields, group-level differences did not reach the established FDR threshold. Notably, the right hemisphere CA2 subfield demonstrated a statistically significant mean difference, indicating a potential region-specific structural alteration. The magnitude and direction of this effect, while requiring cautious interpretation, may reflect underlying neuropathological processes localized within the CA2 area that could be associated with schizophrenia. This finding provides robust evidence for the anatomical specificity of hippocampal alterations and underscores the importance of sub-regional analyses, as broader hippocampal measures may obscure subtle changes.

CA2 is probably the most enigmatic of the hippocampal subfields (42). The studies on physiological properties and behavioral correlates of CA2 demonstrated that this small subregion has remarkably distinct properties compared to the rest of the hippocampus. The unique connectivity and physiological properties of CA2 pyramidal cells make this region a computational hub at the core of hippocampal information processing (43). One of the important hypotheses in the etiopathogenesis of schizophrenia is changes in the regulation of kainate-sensitive glutamate receptors (kainate receptors) in the hippocampus. An important finding was that a post-mortem study testing this hypothesis reported that the decrease in GluR_5,6,7_ immunoreactivity density in apical dendrites in the stratum radiatum and stratum lacunosum-moleculare compared with healthy controls was more pronounced in CA2 than in CA3 or CA1 (44).

The current findings, indicating a selective volumetric difference in the right hemisphere CA2 subfield, underscore the value of examining discrete hippocampal subfields rather than relying solely on whole-structure metrics. Although most left and right hemisphere subfields did not yield significant differences after stringent FDR adjustments, the CA2 alteration suggests a potential localized neuropathological process in schizophrenia. While these findings generally resonate with existing literature that highlights hippocampal alterations in schizophrenia, the specific involvement of the right CA2 subfield presents a more nuanced pattern than some previous reports, which have frequently emphasized other hippocampal subfields. In an early investigation employing stereotaxic space and surface-based mesh modelling, the mid-to antero-lateral hippocampal regions displayed pronounced volumetric alterations accompanied by corresponding increases in peri-hippocampal cerebrospinal fluid in first episode schizophrenia (45). In another study employing both FreeSurfer v5.1 and manual segmentation methods—each yielding highly correlated findings—individuals with schizophrenia or schizoaffective disorder exhibited reduced hippocampal volumes relative to controls when examining the whole hippocampus (46). In a study using FreeSurfer 6.0 and its development version, several hippocampal subfields, including bilateral presubiculum and molecular layer, the left hippocampal tail, subiculum, and CA1, as well as the right parasubiculum, have been reported to show smaller volumes in patients with schizophrenia (47). In a study employing FreeSurfer 5.3 and MAGeT (48), chronic patients demonstrated bilateral volume reductions in CA4/DG, CA2/CA3, and the stratum, as well as decreased volume in the right subiculum when compared to older healthy controls. However, no subfield volume differences were observed between recent-onset patients and younger healthy controls in either hemisphere (49). In a recent study employing the Automated Segmentation of Hippocampal Subfields software with the Penn PMC atlas (50), individuals in the early stages of psychosis exhibited lower volumes in the anterior CA1 and DG subfields compared to healthy controls, while no differences were observed in CA2/3 or the subiculum. A more recent investigation reported that volume deficits in CA1 and the presubiculum were evident at baseline, and that atrophy extended to the GC/ML/DG and CA4 by week 16 (51). Schizophrenia’s underlying heterogeneity may partly explain why our findings differ from previous reports. Nonetheless, by enabling researchers to analyze brain MRI scans with unprecedented granularity (29), NextBrain allows for more detailed and region-specific insights. Its application here may have facilitated the detection of the selective right CA2 volumetric alteration, thereby providing a more refined understanding of hippocampal involvement in schizophrenia.

This study is subject to several limitations. First, its cross-sectional design precludes drawing conclusions about the longitudinal trajectories of hippocampal subfield changes and their potential causal roles in schizophrenia. Second, although the ComBat algorithm reduced site-related variability, residual differences in MRI acquisition parameters and scanner characteristics may still influence volumetric measures. Third, while the NextBrain segmentation tool is robust and fine-grained, it relies on probabilistic atlases and histological references that may not fully capture inter-individual anatomical variability. Fourth, the absence of direct histological validation constrains the interpretability of the detected sub-regional alterations. Moreover, the predominance of antipsychotic treatment in our patient cohort; with only 4.9% of patients being drug-naive, raises concerns that medication effects might influence the associations between diagnosis and hippocampal morphology. However, some evidence suggests that pre-treatment hippocampal enlargements can return to normal following antipsychotic therapy (52). Furthermore, in a study, initial volume deficits in CA1 and the presubiculum at baseline expanded to include the molecular and granule cell layer of the dentate gyrus (GC/ML/DG) and CA4 by week 16 with a risperidone treatment (51). Additionally, reliance on existing data repositories may introduce selection biases or other unforeseen confounders. Finally, although the “full” NextBrain processing approach can technically be executed using CPU-based methods, its reliance on advanced GPU capabilities may limit reproducibility and scalability in resource-constrained settings. Since the inception of this manuscript, however, a “fast” version of NextBrain has been introduced, wherein atlas deformation is pre-computed via a neural network and remains fixed throughout the optimization process, thereby reducing computational demands (31). Finally, IHI is more common in schizophrenia compared to healthy controls, and the presence of IHI may affect segmentation accuracy (25, 26, 53). Our segmentation method may have been inadequate in characterizing IHI.

Evidence indicates that volumetric deficits originate in the CA1 subfield during the early stages of the illness and then extend to other hippocampal regions as schizophrenia progresses (24, 54). Conversely, other evidence suggests that hippocampal volume loss peaks in chronic schizophrenia, without clear progression within the first two to five years of illness (12). Given that our cohort (MCICShare) had a median illness duration of seven years, and the cross-sectional design of our study our findings neither confirm nor refute these trajectories, yet their unexpected nature does not render them unprecedented. Taken together, these observations, alongside our own results underscore the substantial heterogeneity within the psychosis population, a complexity that may be more thoroughly elucidated through the fine-grained, detailed analyses afforded by NextBrain.

## 5 Conclusions

Considering our study limitations and the novelty of the 3D histological mapping method, our results support the value of high-granularity segmentation approaches and raise new questions about the specific role of CA2 alterations in schizophrenia’s pathophysiology, potentially guiding future research and clinical applications.

## Data Availability

The raw data supporting the conclusions of this article will be made available by the authors, without undue reservation.
