# Supplementary material for: 3D histological mapping of hippocampal subfields: a comparative study in patients with schizophrenia and healthy controls
Source: Front Psychiatry. 2025 Oct 6;16:1682782. doi: 10.3389/fpsyt.2025.1682782 (PMC12535999; doi:10.3389/fpsyt.2025.1682782)
Supplement: Supplementary file 1 [file Supplementaryfile1.pdf]

Supplementary Document

ST 1. Assumption Checks of the Model: $Diagnosis \sim CA2 + eTIV + age + sex$

Test/Plot		***p***
**Homogeneity of Variances Test (Levene's)**	F = 1.98	0.119
**Normality Test (Shapiro Wilk)**	Statistic = 0.992	0.366
**Q - Q Plot**	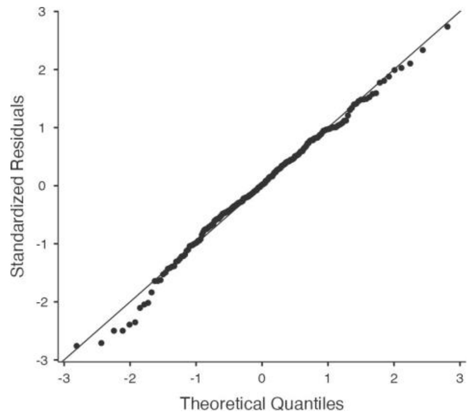	

ST 2. Characteristics of the Participants of the MCICShare Study Between Sites

Variable	**Site A** n = 88	**Site C** n = 56	**Site D** n = 58	**test**	**p**	**Effect size**
**Age**	27 [22 - 39]	28 [24 - 41]	42 [34.3 - 46]	20.1*	<.0001	0.100†
**Sex**, *female*	19 (21.6%)	19 (33.9%)	18 (31.0%)	3.05‡	0.218	–
**Diagnosis**, *Schizophrenia*	44 (50%)	31 (55.4%)	33 (56.9%)	0.78‡	0.677	–

Note. Data are presented as median [25th percentile - 75th percentile] for continuous variables and number (percentage) for categorical variables. Percentages represent the proportions within the site.

* Kruskal-Wallis χ^2 value,† ε^2 value, ‡ Contingency tables χ^2 test
